# Effects of Solids Retention Time on the Anaerobic Membrane Bioreactor with Yttria-Based Ceramic Membrane Treating Domestic Wastewater at Ambient Temperature

**DOI:** 10.3390/membranes10090196

**Published:** 2020-08-21

**Authors:** Rathmalgodage Thejani Nilusha, Dawei Yu, Junya Zhang, Yuansong Wei

**Affiliations:** 1 State Key Joint Laboratory of Environmental Simulation and Pollution Control, Research Center for Eco-Environmental Sciences, Chinese Academy of Sciences, Beijing 100085, China; nthejani@yahoo.com (R.T.N.); dwyu@rcees.ac.cn (D.Y.); jyzhang@rcees.ac.cn (J.Z.); 2 Environment Technology Section, Industrial Technology Institute, 363, Bauddhaloka Mawatha, Colombo 07 00700, Sri Lanka; 3 Department of Water Pollution Control Technology, Research Center for Eco-Environmental Sciences, Chinese Academy of Sciences, Beijing 100085, China; 4 University of Chinese Academy of Sciences, Beijing 100049, China; 5 Institute of Energy, Jiangxi Academy of Sciences, Nanchang 330029, China

**Keywords:** AnCMBR, solids retention time, microbial community, foulants, ambient temperature

## Abstract

The effects of solid retention times (SRTs) (100 days, 50 days, 25 days) on the performance, microbial community, and membrane fouling of a lab-scale anaerobic yttria-based ceramic membrane bioreactor (AnCMBR) treating synthetic domestic wastewater at ambient temperature (31.2 ± 2.7 °C) were examined. The soluble chemical oxygen demand (SCOD) removal was higher (89.6%) at 25 days SRT compared with 50 days (39.61%) and 100 days (34.3%) SRT. At 100 days SRT, more *Bacteroidetes, Firmicutes,* and *Proteobacteria* were present in the microbial community. At 25 days SRT, more *Chloroflexi, Synergistetes,* and *Pastescibacteria* emerged, contributing to the stable performance. The SRT of 25 days has resulted in a more stable microbial community compared with 50 days and 100 days SRT. Both bacterial and archaeal community diversities were higher at 25 days SRT, and the specific production of soluble microbial by-products (SMPs) and extracellular polymeric substances (EPSs) were higher at 25 days SRT as well. Consequently, the membrane flux was lower at 25 days SRT with the increased particle size and the enhanced SMPs and EPSs production. Fourier transform infrared spectroscopy analysis (FTIR) and three-dimensional excitation and emission matrix (3D-EEM) analysis showed that protein and SMPs were the major membrane foulants at all SRT stages. In this study, SRT at 25 days was favorable for the stable operation of an AnCMBR treating domestic wastewater at ambient temperature.

## 1. Introduction

The anaerobic membrane bioreactor (AnMBR) technology provides a lucrative solution for domestic wastewater treatment [1]. AnMBR merges the anaerobic biological wastewater treatment process with membrane technology [2]. It reduces energy consumption and sludge production compared to conventional aerobic processes MBRs [3]. AnMBR applications on domestic wastewater (DWW) treatment can help combat droughts by reusing the wastewater and nutrients in agriculture, especially in tropical countries [4]. Anaerobic treatment has been successful for treating DWW in tropical countries at an ambient temperature of around 20–35 °C considering the cost reduction for heating to maintain mesophilic conditions [5]. Nevertheless, still, better process optimization is necessary for AnMBR to operate at ambient conditions. There is a great lacuna of research and application of ambient temperature AnMBR for DWW treatment and reuse, notwithstanding its so-called opportunities over thermophilic and mesophilic operation.

The bioprocess efficiency of anaerobic process is determined by solid retention time (SRT), hydraulic retention time (HRT), and organic loading rate (OLR) [6]. AnMBR enables the decoupling of SRT independent of HRT [7]. Skouteris et al., 2012 reported that AnMBR was usually operated at SRT longer than 150 days, while HRT ranged from 2 h to 20 days [8]. For the better process optimization of AnMBR, anaerobic bacteria require longer SRTs. Therefore, the maintenance of long SRT is common in AnMBR, allowing slow-growth biomass to be retained in the bioreactor, which is one of the challenges of anaerobic treatment [9]. On contrary, decreasing SRTs push the degradation capacity of anaerobic microorganisms [10]. Similarly, at short SRT, the biogas production is enhanced due to high volatile solids destruction efficiency that gives a positive energy balance for the process. [11]. Obviously the short SRT operation encourages efficient anaerobic treatment utilizing a small footprint [10]. Therefore, the novel approach of this study was to decrease the SRT, leaving the other operational conditions unchanged at the ambient conditions.

Likewise, understanding the effects of SRT on the microbial community is crucial to achieve excellent performance whilst optimizing the process of an AnMBR [12]. Limited studies have investigated the microbial community composition and dynamicity of AnMBR at ambient temperature treating domestic wastewater [13,14,15,16]. Thus, this study focused on ambient temperature microbial community shifts under varying SRTs of anaerobic yttria-based ceramic membrane bioreactor (AnCMBR) operation.

The other significant aspect of AnMBR operation is membrane fouling [17,18]. The effect of SRT on membrane fouling is important in AnMBR stable operation. The correlation between SRT and membrane fouling in an AnMBR was previously studied [19]. Increasing the SRT has increased membrane fouling in an AnMBR [20,21]. The longer the SRT, the higher the particle deposition on the membrane surface [20]. The impacts of SRT on an AnMBR with polymeric membranes for treating domestic wastewater at mesophilic and thermophilic conditions were well discussed [22]. Nowadays, ceramic membranes for AnMBR systems are demanded due to their unique advantages over polymeric membranes such as high surface hydrophobicity, good mechanical and chemical stability, etc. [23]. Ceramic membranes are made of a mixture of diverse mineral oxides (Al_2_O_3_, ZrO_2_, TiO_2_, and SiO_2_) [24]. Previously, alumina-based ceramic membranes have been successfully applied in AnCMBR for domestic wastewater treatment [25]. Yet, these membrane materials showed considerable fouling. Novel composite ceramic membranes are further beneficial to reduce membrane fouling. Accordingly, the other novel approach of this study was the application of a yttria-based ceramic tubular membrane with a good antifouling ability. Yttria (Y_2_O_3_) impregnation had shown a significant reduction of biofouling [26] and high resistivity to higher temperature [27].

Therefore, this work evaluated the effects of SRT on the (i) bioprocess performance, (ii) membrane fouling, and (iii) microbial community at the ambient temperature operation of AnCMBR for DWW treatment. The findings will be helpful in the future design of an AnCMBR and process optimization for tropical areas.

## 2. Materials and Methods

### 2.1. Bioreactor and Start-Up

**Figure 1:**
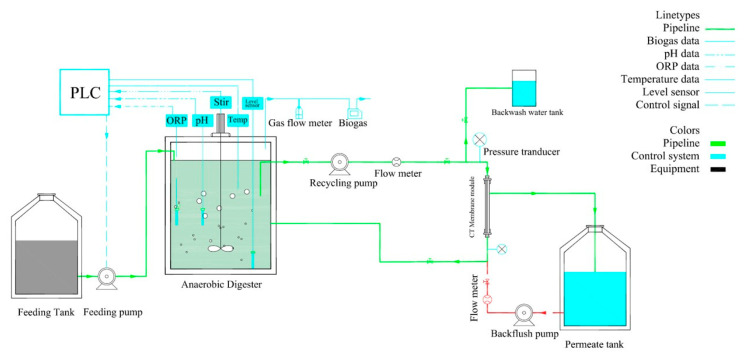
The schematic diagram of the setup.

A continuous stirred tank reactor (CSTR) made of plexiglass with a working volume of 15 L (Diameter × Height = 120 mm × 650 mm) used in our previous study [28] was used at laboratory scale (Figure 1) under different operational conditions. A level sensor (AF-E2A3C1D1B2), pH, and oxidation/reduction potential (ORP) probe (ACTEON5000, PONSEL group, Aqualabo Analysis, Caudan, France) were placed inside the reactor. The reactor was connected to an external ceramic mono tubular microfiltration membrane made of ceramic composite, yttria/zirconia with special antifouling ability (Hefei ShiJie Membrane Engineering Co., Ltd, Hefei, China) (Pore size × Filtration area = 100 nm × 0.011 m^2^, Length 50 cm, in-out orientation). The anaerobic reactor was fed with synthetic domestic wastewater (Section 2.2) using a peristaltic pump (BT100-1L, Longer, YZ1515x Pump, Baoding, China). A Xin Xishan DP-35 diaphragm pump (Xin Xishan industries Co., Ltd, Shanghai, China) was used for membrane feeding and recirculation. The backwash pump (25WZR-15, Xin Xishan industries Co., Ltd, Shanghai, China) was used for backwashing once a day for 60 s based on [28]. A programmable logic controller (PLC) (LAB VIEW, PLC, Siemens AG, Frankfurt, Germany) automatized the setup operation. Figure S1 shows this automatic control strategy. A biogas flow meter (U-Flow, Bioprocess Control AB, Stockholm, Sweden) was used for monitoring biogas production. A flow meter (NRLD-20, Ruiji automation company, Nanjing, China) was used to record the membrane flux. The reactor was inoculated with anaerobic sludge acquired from the Gao’an’tun wastewater reclamation plant (Beijing, China) during the startup. Nitrogen gas was purged during inoculation. The AnCMBR was operated at different SRTs, as shown in Table 1. A small volume of secondary inoculum after 50 days was used from a lab-scale AnMBR treating potato starch to keep sustainable microbial community in the reactor. The microbial community of this inoculum was more or less similar to the previous inoculum (Figures 7 and 8).

### 2.2. Domestic Wastewater Characteristics

Synthetic domestic wastewater was prepared to represent domestic wastewater according to our previous work [29]. Its composition is listed in Table 2. Glucose and sodium acetate were the carbon sources, and ammonium chloride was the nitrogen source. Potassium dihydrogen phosphate was applied as the phosphorus source. The influent C/N ratio was adjusted to 5:1 based on [30], as carbon and nitrogen are the vital nutrients and source of energy for the growth of microorganisms [29]. This ratio is important for the microorganisms to function at maximum efficiency, resulting in a stable anaerobic digestion process [28,29,31]. Additionally, the essential trace metals (0.17 g H_3_BO_3_, 1.52 g FeCl_3_.6H_2_O, MnCl_2_.4H_2_O 0.15 g, CoCl_2_.6H_2_O) (1 mL/L) were supplied according to Zhang et al., 2018 [32].

### 2.3. Analysis of Physico-Chemical Parameters

Samples from the reactor, influent, and permeate were sampled twice a week for the analysis of mixed liquor suspended solids (MLSS), mixed liquor volatile suspend solids (MLVSSs), soluble chemical oxygen demand (SCOD), total alkalinity, and volatile fatty acids (VFAs). SCOD was measured using a spectrophotometer (DR6000, HACH Inc., Loveland, CO, USA) following standard methods (APHA). MLSS and MLVSS were determined at 104 °C (4 h) and 600 °C (2 h), respectively. VFAs (acetic, propionic, *i*-butyric, *n*-butyric, *i*-valeric, and *n*-valeric acids) were measured using a GC-2014C Shimadzu gas chromatograph with Shimadzu AOC-20i Auto injector (Kyoto, Japan). Alkalinity was measured using a HACH TNT plus^TM^ 870 Total alkalinity test kit (25–400 mg/L as CaCO_3_) (Loveland, CO, USA). Particle size distribution (PSD) of inocula and the anaerobic sludge was analyzed using a size exclusion chromatography (Malvern Mastersizer 2000, Malvern Instruments Ltd., Worcestershire, UK). pH and oxidation reduction potential (ORP) was daily recorded using online oxidation/reduction potential (ORP) probe (ACTEON5000, PONSEL group, Aqualabo Analysis, Caudan, France).

### 2.4. Microbial Community Analysis

Samples from two inocula and anaerobic sludge representing different SRTs on Day 1, 15, 30, 45, 60, 75, 90, 110, 125, and 150, respectively, were analyzed for bacterial and archaeal community composition. The DNA of these samples was extracted using a FAST DNA Spin Kit for Soil (MP Biomedicals, Solon, OH, USA) according to the manufacturer’s instructions. Bacterial and archaeal diversity was evaluated by PCR amplification of 16S rRNA genes using the 515FmodF_806R and silva_Arch349F-Arch806R primers following the procedure by Lu et al., 2019 [33]. Sequencing was conducted at the Sangon Co., Ltd (Shanghai, China).

### 2.5. Membrane Fouling Analysis

Transmembrane pressure (TMP) and flux were daily recorded. A Fourier transform infrared spectroscope (Nicolet–iZ10, Thermo Scientific, Waltham, MA, USA) was used for the analysis of major foulants in the mixed liquor. First, 5 mL of mixed liquor was freeze dried for 48 h and then smashed as powder for Fourier transform infrared spectroscopy analysis (FTIR) analysis. The extracellular polymeric substances (EPSs) analysis was used to determine whether the fouling originated from the protein or carbohydrate of EPS fractions following the methods suggested by [34]. Proteins and carbohydrates were analyzed using a UV-vis spectrophotometer (TU-1901, Shaanxi, China)) at a wavelength of 750 nm and 480 nm, respectively. Organic foulants of the extracted EPS and soluble microbial by-products (SMPs) were characterized and analyzed using a three-dimensional fluorescence excitation–emission matrices analyzer (3D-EEM, F-7000, Hitachi, Tokyo, Japan). After 150 days running, the fouled membrane module was dismantled from the reactor unit and ex situ chemical cleaning was conducted. Chemical cleaning sequence included the following: (1) permeate cleaning, then soaking in pure water for 8 h; (2) cleaning with NaOCl at effective Cl^−^ concentration of 500 mg/L followed by soaking in pure water for 8 h; and (3) cleaning with 500 mg/L citric acid solution followed by soaking in pure water for 8 h. Ceramic membrane autopsies were not used for fouling analysis, as the cleaned membrane was used for a further filtration process. Cleaning solutions were subjected to 3D-EEM analysis and microbial community analysis in order to identify major organic and microbial foulants, which will be described in a future publication.

### 2.6. Statistical Analysis

Statistical analysis was performed using MINITAB 14 software (Minitab Inc., State College, PA, USA) for one-way ANOVA analysis and principal component analysis (PCA). The microbial community analysis (community abundance bar plots, heat map, redundancy analysis (RDA)) were performed using the free online platform of Majorbio Cloud Platform (www.majorbio.com), Shanghai Majorbio Bio-pharm Technology Co., Ltd, Shanghai, China.

## 3. Results and Discussions

### 3.1. Impact of SRT on AnMBR Performance

#### 3.1.1. COD Removal

**Figure 2:**
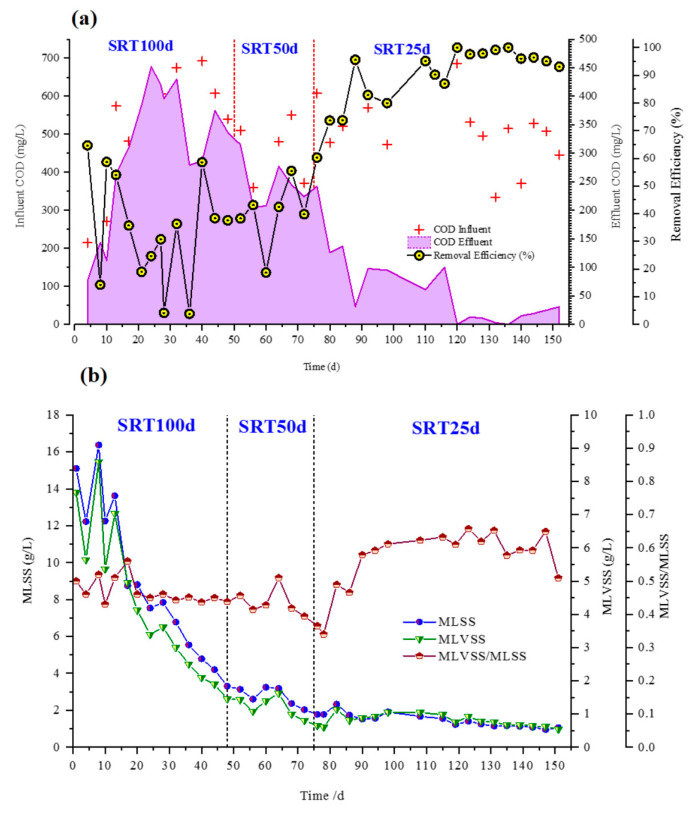
The variation of AnCMBR performance parameters (**a**) soluble chemical oxygen demand (SCOD) removal (**b**) MLSS, MLVSS, and MLVSS/MLSS ratio.

As shown in Figure S2, the reactor was operated at the ambient temperature (31.2 ± 2.7 °C). However, 36 °C has been recorded as the highest temperature, which deviated from this ambient temperature range. It was due to the prevailed local high temperature during the summer in Beijing. During ambient temperature operation, such deviations occur due to seasonal and diurnal temperature variations [35]. However, the summer temperature in Beijing is more or less similar to that of the tropical temperature in Sri Lanka. In this study, a water bath was not used as a means of cost cutting and to simulate the real ambient conditions, as our pilot studies are expected to be conducted in Sri Lanka. Figure 2a shows the soluble chemical oxygen demand (SCOD) removal during the operation. Generally, the influent SCOD concentration ranged from 400 to 600 mg/L. The synthetic influent was daily prepared and added to the influent tank. However, due to the readily biodegradable nature of glucose and the diurnal variation of temperature effects on biodegradability, a great variability of SCOD resulted in the influent in some occasions. SCOD removal has varied with different SRTs. In the first operational period when the SRT was 100 days, considerably lower removal efficiencies were observed compared to 50 and 25 days of SRTs. At 100 days SRT, the average permeate SCOD concentration was 305 ± 120.69 mg/L, corresponding to an SCOD removal efficiency of 34.3% ± 19.82. Bandara et al., 2012 also reported very low SCOD removal at ambient conditions for DWW treatment [15]. This might be due to the high concentration of hydrolyzing/fermenting bacteria at long SRTs. Their high abundance can limit the substrate for methanogens, further reducing the methane production rate and resulting in low organic matter removal [36]. Another reason for less SCOD removal can be the reduction of microbial activity due to the shear force of the high-speed recycle pump [21]. SRT was reduced to 50 days during Days 56–75. Thereby, the SCOD removal has slightly increased to 39.61% ± 11.89. Then, SRT was further reduced to 25 days during Days 76–150 to elucidate the effect of decreasing SRT. As shown in Figure 2a, a significantly higher mean SCOD removal of 89.6% ± 11.4 than the other two SRT stages were achieved in this stage. The average permeate SCOD concentration became less than 50 mg/L at the steady state of 25 days SRT adhering to both Chinese (GB 18918-2002) and Sri Lankan standards for treated DWW discharge.

#### 3.1.2. MLSS and MLVSS

Figure 2b illustrates the variation of the mixed liquor concentration (MLSS, MLVSS, and MLVSS/MLSS ratio) at different SRTs. The MLSS or MLVSS concentrations in the AnMBR were found to decrease with the shortening of SRT. One reason could be the continuous sludge discharge from the reactor to maintain the short SRT. However, the MLVSS/MLSS ratio has increased (0.58 ± 0.12) at an SRT of 25 days compared with the other two SRTs. These disparities can be partly due to the enhanced activity and dominancy of methanogens at short SRTs (described in Section 3.3.1 and Section 3.3.2). Yeo and Lee 2013 encountered similar findings with this study: less concentration of active methanogens at SRT 40 days than at SRT 20 days [36]. At 25 days SRT, the MLSS concentration in the bioreactor stabilized at around 1.8 g/L, resulting in maximum SCOD removal. Maintaining low MLSS concentration is advantageous, as high MLSS concentrations at longer SRTs increases the viscosity, which makes filtration and sludge agitation difficult. Some previous CSTR coupled with membrane filtration has also been successful at low MLSS levels [37]. Kocadagistan and Topcu 2007 have also reported MLSS = 1.05–2.41 g/L for municipal wastewater treatment with CSTR with a flat polymeric membrane in AnMBR [38]. This study also followed the general practice in AnMBR operation, which is to start with a long SRT to establish and adapt the stable microbial community and then slightly reduce the SRT based on the reactor performance [19]. However, if this experiment was switched in to a short SRT operation first and then a longer SRT, the results can be different. Huang et al., 2011 conducted their experiment in increasing order of SRTs (30 days, 60 days, and infinite). It yielded an increase of MLSS with an increase of SRTs in agreement with our experiment, although the experiment order was reversed [39]. Another study by the same author was also in agreement with this [21]. However, more studies should be conducted to confirm this interpretation.

#### 3.1.3. VFA and Alkalinity

The acidification index (total volatile fatty acids/total alkalinity) (VFAs/ALK) is regarded as an early-warning indicator of acidification in anaerobic treatment system. Generally, the desirable VFAs/ALK ratio in anaerobic bioreactors should be less than 0.3 [29]. Figure S3 exhibits the variation of VFAs (acetic, propionic, *i*-butyric, *n*-butyric, *i*-valeric, and *n*-valeric acids) and VFAs/ALK ratio. At 100 days SRT, the acetic acid concentration varied between 920 and 200 mg/L. At 50 days SRT, there was no significant reduction of the VFAs accumulation. For stable performance of the anaerobic reactor, the VFAs concentration should be between 50 and 250 mg/L [40]. At SRT 100 and 50 days, the VFAs/ALK ratio was very much higher than the critical value of 0.3, indicating the instability. The VFAs accumulation may be a consequence of the slow biomass synthesis and reduced methanogenic microorganism activity [41]. Furthermore, the accumulation of long-chain fatty acids (LCFAs) in the system is directly related to the SRT. The higher the SRTs, the higher the chances of accumulating LCFAs due to the reduced wastage of these compounds with the sludge wasting. Derali et al., 2014 reported a severe LCFA inhibition on the biological performance and methanogenic activity when working at 50 days SRT when treating corn-to-ethanol thin stillage [42]. This accumulation of LCFAs can be linked with the reported low MLSS concentration. The reduced SRT to 25 days provided the lowest accumulation of VFAs and the lowest VFAs/ALK ratio ratio of <0.3, indicating stable process performance in the system. The low VFA concentration is an indicator of the good balance between acidogenesis, acetogenesis, and methanogenesis processes. This denotes the enhanced efficiency of methanogens as further convinced by microbial community analysis. Apart from the VFAs/ALK ratio, the total alkalinity has varied with varying SRT. During stages at SRTs of 100 days, 50 days, and 25 days, the average alkalinity values were 519 ± 127.48, 490 ± 126.98, and 506 ± 273.20, respectively. This is well lower than the general alkalinity requirement for anaerobic systems, which is 2000–4000 mg CaCO_3_/L [43]. This could be attributed to the nature of the influent wastewater or to the low temperature. As an example, our previous study by Martin et al., 2018 has resulted in alkalinity 675 ± 188.42 mg/L CaCO_3_, which was sufficient for sustaining the anaerobic microbial community with the same synthetic wastewater [29]. Moreover, as mentioned in the following section, due to the membrane feeding pump failure that occurred on Day 85, the significant changes in alkalinity have been reported as the ANOVA analysis of SRT versus alkalinity resulted in P values less than 0.05. Although the alkalinity showed a deficiency in the system, the VFAs/ALK ratio indicates an efficient and stable anaerobic digestion process. The relative ANOVA analysis for SRT versus TVFA showed no significant relationship for both with and without the aforesaid pump failure. However, alkalinity optimization was not considered in this study. This scenario and the deficiency of alkalinity in AnMBR at the ambient temperature should be further evaluated in future studies for treating domestic wastewater [43].

### 3.2. Effect of SRT on Membrane Fouling

#### 3.2.1. TMP and Flux Evolution

The TMP, flux (a), permeability, and cross flow velocity CFV (b) variation in this study are shown in Figure S4. The mean TMP at 100 days of SRT was 73.33 ± 2.01 kPa, while the flux was 50.82 ± 4.39 Lm^−2^ h^−1^. At an SRT of 50 days, the mean TMP was 75.22 ± 1.18 kPa, while the flux was 49.68 ± 2.38 Lm^−2^ h^−1^; at 25 days SRT, the mean TMP and flux was 76.54 ± 4.94 kPa and 34.40 ± 13.46 Lm^−2^ h^−1^, respectively. After 85 days and 103 days, a recycling pump failure was encountered. A rapid increase of the TMP was encountered, corresponding to that failure, and with the second failure, the flux was rapidly declined to below 20 Lm^−2^ h^−1^. The permeability has also shown considerable reduction over the time corresponding to aforesaid variations in TMP and flux. To determine whether there is a significant difference between the means of flux and TMP under studied SRTs, one-way ANOVA followed by Tukey’s post hoc analysis was carried out using Minitab 14 software. Obtained p-values in all incidences were less than 0.05, denoting that the means are significantly different; thereby, SRT has affected the membrane fouling behavior. Furthermore, in order to show the effect of pump failure on the relationship between SRT and flux and TMP variation, those data after 85 days were omitted, and ANOVA analysis was conducted. The obtained p-value for the relationship between SRT versus TMP was 0.00 whereas it was 0.038 for SRT versus flux. Therefore, after omitting the pump failure also, a significant relationship existed in SRT versus flux and SRT versus TMP. Furthermore, referring to the flux after fixing the pump failure at 85 days, it was 20 Lm^−2^ h^−1^. However, it became stable, resulting over 50 Lm^−2^ h^−1^ in the following days and it lasted until the next pump failure. Therefore, this pump failure on Day 85 did not lead to an adverse non-reversible fouling, which altered the membrane permeability. Two other pump failures on Day 97 and Day 123 also showed that the flux after the failure can be recovered to a stable and higher value, indicating that corresponding flux declines are mainly due to the changes in the pump and related pressure. These pump failures have greatly altered the integrity of the experiment, and it is recommend to have a better selection of membrane feeding pumps to achieve sustainable membrane filtration.

#### 3.2.2. Sludge Particle Size Distribution

**Figure 3:**
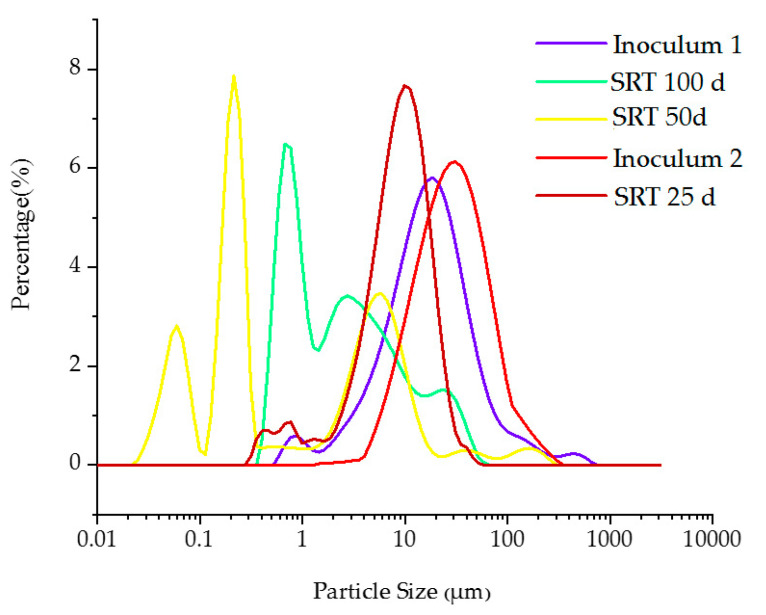
The sludge particle size distribution of the AnCMBR at different SRTs.

Sludge PSD at different SRTs along with the inoculums is shown in Figure 3 characterized by a normal distribution. The PSD of the inoculum 1 is characterized by a uni-model distribution with the mean particle size of 18.7 µm. After 25 days at SRT 100 days, PSD distribution showed a tri-model distribution with three significant peaks at 0.67 µm, 2.75 µm, and 24.1 µm. Similarly, at SRT 50 days also, a tri-model distribution with another three significant peaks at 0.059 µm, 0.214 µm, and 5.92 µm, respectively, were appeared. However, the mean particle size has reduced when the SRT was reduced. The inoculum 2 was added after 50 days, which also showed a uni-model distribution with the mean particle size of 31 µm. Compared to the reactor’s working volume (15 L), the applied volume of the second inoculum applied was very small volume (>1 L). When the SRT was further reduced to 25 days, the sludge particle size increased, and the shape has become a uni-model distribution with the mean particle size of 9.86 µm. Huang et al., 2013 also reported an improved particle size at shorter SRTs [21]. This increase of particle size is attributed to the enhanced biomass activity at 25 days SRT. If the particle size of the anaerobic sludge is larger than the size of the membrane pores, the deposition of this particle on the membrane surface can occur, contributing to the growth of a cake layer. If the particle size of the anaerobic sludge is smaller than the size of the membrane pore, particles can enter the membrane pore and block the pores. In this study, the membrane pore size was 0.1 µm. However, the average particle size in the anaerobic sludge was over 0.1 µm in all three SRT stages. Thus, the cake layer formation might be the dominant fouling mechanism. Furthermore, the cake layer resistance was calculated, and it will be reported elsewhere in a future publication.

#### 3.2.3. SMPs and EPSs

**Figure 4:**
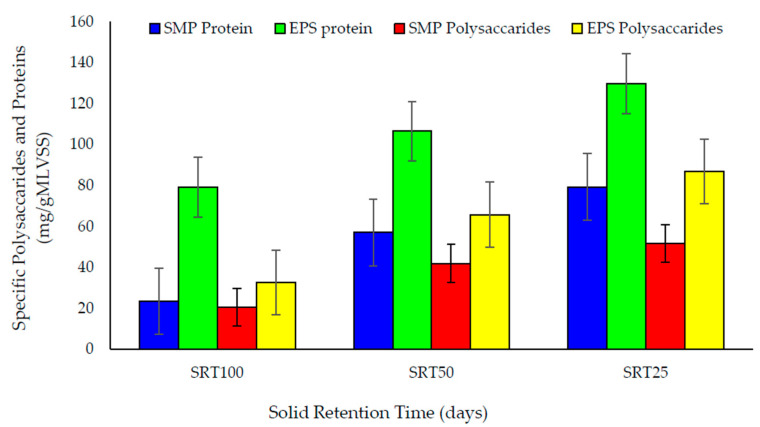
Specific production of soluble microbial by-products (SMPs) and extracellular polymeric substances (EPSs) at various SRTs.

SMPs and EPSs greatly impact the membrane fouling [44]. The specific production of SMPs and EPSs expressed by protein and carbohydrate production per gram MLVSS is shown in Figure 4. The soluble state of SMP fractions contributes to pore blocking [45]. The longer the SRT, the lesser the concentration of EPSs [37]. This study showed similar results: low SMP and EPS concentrations over a long SRT, while high SMP and EPS concentrations over a short SRT. The EPS protein and carbohydrates were higher than that of the SMP protein and carbohydrates in all stages. For both SMP and EPS polysaccharides, proteins were more or less similar in all SRT stages, as shown in Figure 4. One-way ANOVA analysis showed no significant difference between protein and carbohydrate. More carbohydrates and proteins at shorter SRT were due to the higher biomass activity [46]. The increase of biomass activity increases the EPS accumulation [47]. In addition, the self-protection behavior shown by microbes increases SMPs [48]. However, the contribution of supernatant SMPs for membrane fouling is reported to range from 17% to 81% [49,50].

As depicted by Figure S4, the flux level has decreased with decreasing SRT, which is related to the increase of SMP and EPS. The reuniting of granular particles can occur at high EPS levels, which increases the particle size [51]. This phenomenon has been effectively confirmed by EPS, SMP, and sludge PSD results in the present study at 25 days of SRT. The greater the amount of EPSs, the more prominent the fouling at shorter SRTs, whereas at high SRTs, the concentration of EPS decreases as the biomass stays longer in the system [19]

#### 3.2.4. Excitation Emission Matrix of Extracted EPS

3D-EEM analysis was performed to (i) understand the contribution of biopolymers for membrane fouling, and (ii) understand the organic matter removal of the reactor on the basis of fluorescence index (FI), biological index (BIX), and humification index (HIX). The results were interpreted as described by Chen et al., 2003 [52]. Figure S5 shows the 3D-EEM spectra of influent, effluent, and extracted EPS and SMP at different SRTs, while Table S1 gives the fluorescence spectral parameters of samples of influent, effluent, and extracted anaerobic sludge. It shows obvious changes in the fluorescence peaks, which are positively correlated with a reducing SRT. The dominant peaks in the influent and effluent samples in all three SRT stages were in region I and region IV substances, indicating the presence of tyrosine-like proteins and SMPs in the bound EPS of the anaerobic sludge region I, II, and IV substances, which were the dominant substances in all SRT stages, while the fluorescence intensity of the peaks in region IV gradually decreased with decreasing SRT. Meanwhile, the peak locations were not strictly affected by SRT, although the intensity has reduced. Therefore, the major contributors to membrane fouling would be region I, II, and IV substances.

**Figure 5:**
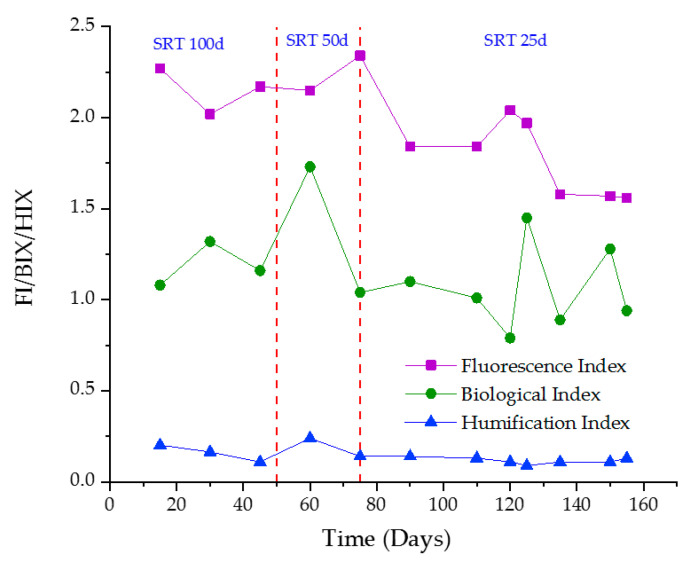
Variations in fluorescence Index (FI), humification index (HIX), and biological index (BIX) values of anaerobic sludge at different SRTs.

3D-EEM spectroscopy not only describes the major organic foulants in the system, but also clearly shows that organic matter transformation/removal was positively correlated with SRT. According to Hudson et al., 2007, the gradually weakening fluorescence intensity has some correlation with SCOD removal [53]. Fluorescence spectroscopy has the potential to describe the humification of dissolved organic matter (DOM). In this study, FI, BIX, and HIX calculated according to [54] were used to examine the effects of SRT on organic matter removal [55]. Figure 5 shows the slight reduction of FI, HIX, and BIX with reducing SRT. HIX provides information on the aromatic degree of the sample. BIX characterizes the maturity of DOM [56]. Thus, the aromatic degree of the system has reduced with reducing SRT. Especially tryptophan and tyrosine-like substances contain aromatic rings. The reduction of HIX denotes that these proteins have been more rapidly degraded into simple organic matter. As a result, the maturity of DOM (BIX) has also reduced. ANOVA analysis was conducted to discern the correlation between variations of FI, BIX, and HIX with that of decreasing SRT. The related *p*-values were greater than 0.05, indicating a non-significant relationship between these indices with SRT. Moreover, the 25 days SRT was omitted, and ANOVA analysis was conducted due to the pump failure at 25 d SRT. Respective *p*-values were greater than 0.05, indicating a non-significant relationship and thus very similar effects with and without the pump failure.

#### 3.2.5. FTIR Analysis

**Figure 6:**
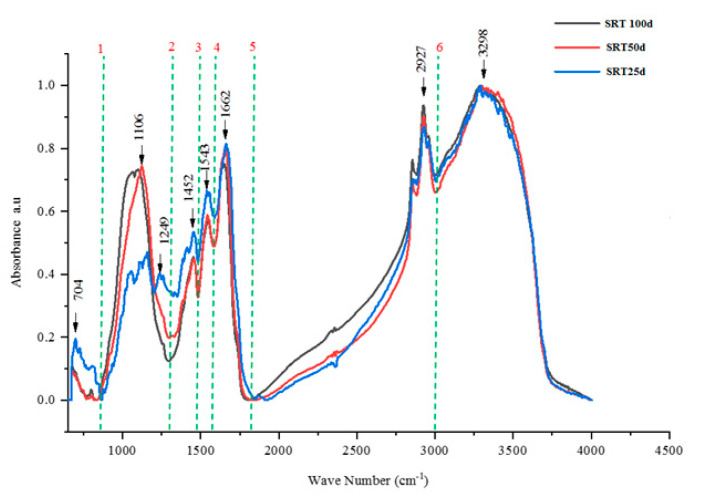
Fourier transform infrared spectroscopy analysis (FTIR) analysis of the anaerobic sludge at different SRTs.

The major functional groups contributing to fouling at different SRTs were observed by FTIR analysis of the anaerobic sludge. As shown in Figure 6, the FTIR spectra indicated eight major peaks at 704 cm^−1^, 1106 cm^−1^, 1452 cm^−1^, 1543 cm^−1^, 1662 cm^−1^, 2927 cm^−1^, and 3298.cm^−1^, respectively. The presence of strengthening of the C–O bond of carbohydrate is given by the broad region of absorbance at 705–1270 cm^−1^. The peaks at 1500–1750 cm^−1^ might be proteins [57]. The peaks at 1452 cm^−1^, 1543 cm^−1^, and 1662 cm^−1^ are related to the protein secondary structure (amide I, II, and III). The absorption bands at or near 2927 cm^−1^ are due to C–H stretching emanating from fatty acids. The broad peak at 3298 cm^−1^ indicates the O–H stretching from hydroxyl functional groups. Accordingly, the FTIR analysis suggests strongly that tyrosine, polysaccharides, proteins, fatty acids, and hydroxyl functional groups should be the dominant foulants in the system during the entire operational period of 150 days, irrespective of the influence of SRT. This is compatible with 3D-EEM results.

#### 3.2.6. Factors of Membrane Fouling

Principal component analysis was conducted to describe the correlation between different fouling factors (MLSS, MLVSS, SMPs and EPSs (protein and polysaccharides) and the different fouling indicators (TMP, flux, permeability, and flux decline coefficient (FDC)) at three different SRTs. Permeability and FDC were calculated according to [58]. Figure S6 shows the results of the principal components’ (PCs) loading plots at three SRT stages. Two PCs were extracted for each SRT. In 100 days SRT, PC2 was positively correlated with biomass characteristics and PC1 was negatively correlated with fouling indicators. FDC was positively correlated in both PCs. At 50 days SRT, biomass characteristics were positively correlated at PC1 and fouling indicators were negatively correlated. At 25 days SRT, permeability and flux were positively correlated in both PCs, while MLSS and MLVSS were negatively correlated. However, EPS polysaccharides were positively correlated in PC2. Increasing EPSs and SMPs increases the fouling at 25 days SRT. EPS and SMP protein and polysaccharides are negatively correlated with flux and permeability.

### 3.3. Evolution of Dominant Microbial Community

**Figure 7:**
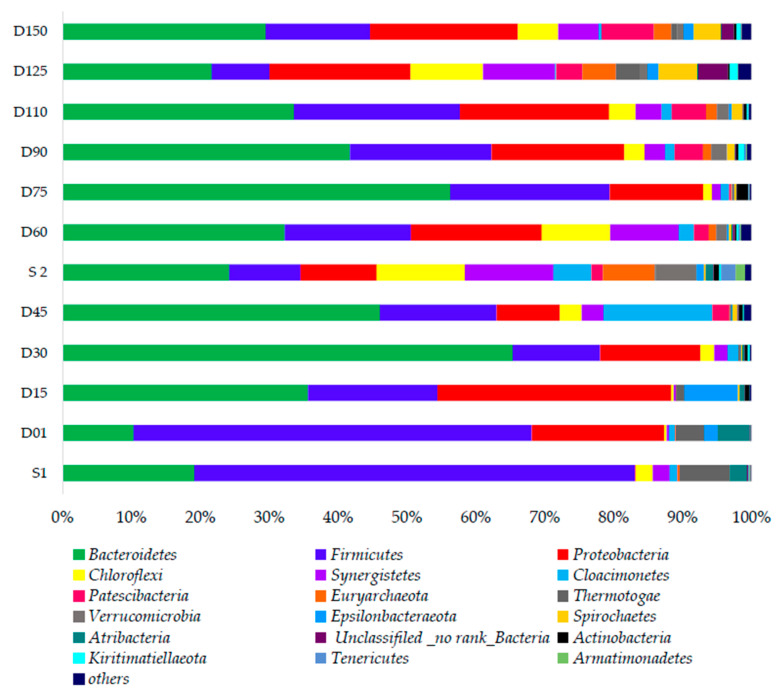
Community bar plot analysis at phylum level bacteria.

**Figure 8:**
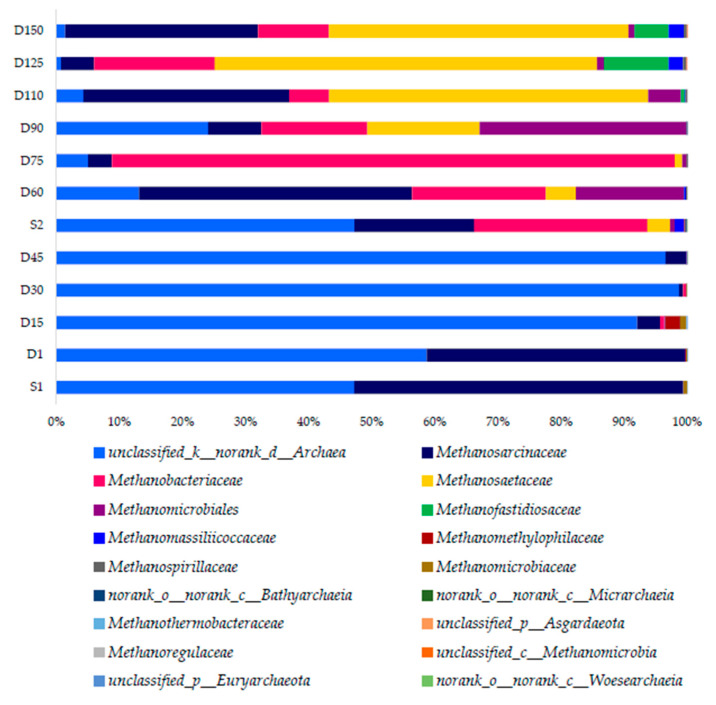
Community bar plot analysis at family-level archaea.

SRT may have a significant effect on the microbial community evolution impacting on biodegradation performance and thus the fouling propensities in MBRs. Figure 7 and Figure 8 illustrate the distribution of the bacterial and archaeal community throughout the operation of the MBRs at varying SRTs.

#### 3.3.1. Bacterial Community Responses

During 100 days SRT, four samples—namely, Day 1, 15, 30, and 45—were analyzed. These samples clearly showed the apparent shifts of the microbial community within similar SRT. During this stage, the commonly appeared phyla were *Firmicutes* (55.7%), *Proteobacteria* (18.5%), and *Bacteroidetes* (9.93%) on Day 01 and *Epsilonbacteraeota* (7.77%) on Day 15. These four phyla have been widely reported in many studies [59,60]. The *Bacteroidetes* phylum plays an important role in cellulose and protein degradation by producing propionate and acetate as products [61]. *Bacteroidetes* have been commonly reported from similar studies such as Cho et al., 2018 in AnCMBR treating food waste recycling wastewater at the ambient conditions [62]. Their abundance could be one reason for the accumulation of acetic acid. *Firmicutes* and *Bacteroidetes* are relevant to the anaerobic hydrolysis and acidification process. The phylum *Cloacimonetes* abundance is significant on Day 45, rating 16.1% of the total abundance. They might be involved in propionate degradation [63]. The dominance of *Firmicutes*, *Proteobacteria*, and *Bacteriodetes* has gradually decreased, and the abundance of *Synergistes* (3.2%), *Patescibacteria* (2.5%), and *Actinobacteria* (0.6%) has increased during SRT 100 days (Day 45). *Actinobacteria* phylum is closely related to biodegradation of aromatic hydrocarbons [64]. The phylum *Thermotogae s*, *Atribacteria*, and *Epsilonbacteraeota* have diminished by Day 15. *Thermotogae* phylum are able to syntrophically oxidize acetate. Their low abundance may be associated with high VFA concentration in the reactor [65]. Then, after 50 days, a second seed was added as mentioned in Section 2.1. The bacterial community on Day 45 and Day 60 with that of seed sludge 2 was compared in order to prove that this introduction has not greatly impacted on the general reactor performance. Both on Day 45 and Day 60, *Bacteroidetes*, *Firmicutes,* and *Proteobacteria* were the three dominant phyla. In addition, the composition of seed 2 is more or less similar to that of Day 45, indicating the presence of *Chloroflexi, Synergistes, Cloacimonetes,* and *Euryarchaeota,* in which the abundance rose later during 25 day SRT. Accordingly, this seed sludge addition might not have altered the performance. Furthermore, at 50 days SRT, *Bacteroidetes* abundance has increased from 34.53% to 57.34%. Similarly, *Firmicutes* abundance has also increased from 19.61% to 23.60%.

During SRT at 50 days, *Proteobacteria* has also become the thirdly dominant bacteria phyla. However, the abundance of *Chloroflexi* has decreased from 10.67% to 1.29%. *Chloroflexi* is involved in the degradation of polysaccharides such as cellulose [63]. During 25 days SRT, four samples were obtained on Days 90, 110, 125, and 150 for the microbial analysis, respectively. There was a slight decrease of the abundance of three major dominant phyla: *Firmicutes*, *Proteobacteria*, and *Bacteriodetes.* Remarkably, the abundance of *Chloroflexi,* (6.1%), *Synergistetes* (6.15%), *Pastescibacteria* (7.92%), *Euryarchaeota* (2.61%), *Thermotogae* (3.8%), and *Spirochaetes* (4.15%) has increased during 125 days. The phylum *Spirochaetes* was found to be a consumer of intermediate metabolites such as glucose. *Synergistetes* were found to be glucose-and acetate-utilizing bacteria [66]. Therefore, the accumulation of VFAs has greatly reduced with the appearance of this phylum at 25 days of SRT. Deeper genus level analysis was further conducted to explore these shifts. Aforementioned changes are clearly compatible with the heat map at the genus level (Figure S7). The genus level also shows that with the decreasing SRT, the abundance of methanogenic genus belonging to *Euryarchaeota* phylum such as *Methanosaeta, Methanobacterium* have emerged in the system. *Methanosaetacea* are acetoclastic methanogens whose high abundance could indicate that the most important pathway to methane production is via acetoclastic methanogenesis, which has further improved SCOD removal. More details on the functions of presented microorganisms are provided in Table S2.

Table S3 shows the diversity indices for bacterial community variation. The Simpson index reflects both the number of species and the evenness of their abundance distribution in a sample. The Chao and Ace estimators emphasize community richness. More precisely, considering the diversity estimator Shannon and richness estimators including Ace and Chao indexes jointly implied that the diversity and richness at SRT 25 days was higher than that of SRT 50 days and 100 days. With the reducing SRT, biomass might have washed out and again stabilized with these dominant aforementioned bacteria phyla at 25 days SRT. In this study, the membrane technology has successfully decoupled a shorter SRT with comparatively longer HRT (48 h) to establish a more diverse anaerobic microbial community. The environmental factor correlation analysis (Figure S8a) was conducted on Majorbio platform and it shows that SRT, TVFA, and ORP have shown the considerable correlation with the abundance of reported bacteria phylum, Especially *Firmicutes* and *Bacteriodetes,* which are in agreement with the other microbial community analysis data.

#### 3.3.2. Archaeal Community Responses

Figure 8 exhibits the family-level community abundance bar plot for archaea. The seed sludge obtained from the partially hydrolyzed sludge of the Gao’antun wastewater treatment plant was abundant with *unclassified_no rank archaea* (47.2%) and family *Methanosaccinaceae* (52.16%). At 100 days of SRT, there was a great dominance of *unclassified_no rank archaea*. The abundance of *Methanosaccinaceae* has greatly reduced to 3.3% on Day 45. This is clearly correlated with the poor SCOD removal and accumulation of VFA at 100 days of SRT. Simultaneously, the SRT was reduced to 50 days. The second seed sludge was comprised of unclassified*_no rank archaea* (47.25%), *Methanosarcinaceae* (19.05%), *Methanobacteriaceae* (27.4%), *Methanosaetacea* (3.54%), *Mathanomassiliicoccaceae* (0.14%), and *Methanomicrobiales* (0.07%). This seed sludge addition has not greatly impacted on the reactor performance. As an example, the archaeal families that became dominant at latter part of 25 days SRT such as *Methanosarcinaceae, Methanobacteriaceae,* and *Methanosaetacea* were also readily available on Day 15. Their abundance was very less during stage of 100 days SRT, which would be attributed to long SRT operation. However, *unclassified_no rank archaea* abundance has further reduced during 60–75 days at 50 d of SRT. *Methanobacteriaceae* (89.2%) has shown a great abundance during 50 d of SRT. At 25 days of SRT, the abundance of *Methanobacteriaceae* has reduced, while *Methanomicrobiales* (32.7%) were promoted at 90 days. *Methanosacinaceae* and *Methanosaetaceae* were the well-adopted families for short SRT conditions at the ambient temperature. *Methanosarcinaceae* and *Methanosaetaceae* have doubling times of 1–2 days and 4–9 days, respectively [67]. Therefore, *Methanosarcinaceae* are generally dominant at shorter SRT. The shift clearly proves the causes for a steep reduction of VFA in the system at 25 day SRT. Furthermore, *Methanobacteriaceae* and *Methanomicrobiales* have a greater maximum growth rate and half-saturation coefficient than *Methanosaetaceae*, leading to the dominance of *Methanosaetaceae* when acetate concentrations are low [68]. Due to this, the abundance of *Methanosaetaceae* (47.49%) has improved at SRT 25 days. Further analysis of the genus level of the archaeal community heat map (Figure S9) also confirmed the aforesaid shifts in genus level. At the genus level, the abundances of *Methanobacterium*, *Thermosesulfobacterium,* and *unclassified c thermopretei* have shown similar shifts. During 25 days SRT of the reactor operation *unclassified Euryarchaeota, Methanobrevibactor* have significantly increased. Table S3 shows the alpha diversity indices during the operational period for archaea community shifts. Accordingly, Shannon and Simpshon diversity indices (Table S4) have jointly shown increasing trends during the 25 days SRT stage. The environment factor correlation analysis (Figure S8b) clearly showed that archaea community abundance has been negatively correlated with SRT, confirming the aforesaid shifts during this study.

## 4. Conclusions

The effects of SRT on the performance of AnCMBR were investigated at the ambient temperature for treating domestic wastewater. The SCOD removal exhibited the highest removal at 25 days SRT compared with 100 days and 50 days SRT, which complied with COD < 50 mg/L (GB 18918-2002) for treated domestic wastewater discharge guidelines. SRT at 25 d resulted in a more stable microbial community compared with 50 days and 100 days of SRT. At the stage of 100 days SRT, more *Bacteroidetes, Firmicutes,* and *Proteobacteria* were present. At the stage of 25 days SRT, more *Chloroflexi, Synergistetes*, and *Pastescibacteria* emerged, contributing to stable performance. The methanogen families *Methanosacinaceae* and *Methanosaetaceae* were also predominant. Methanogens were less abundant at 50 and 100 day SRTs. FTIR and 3D-EEM analysis showed that the major membrane foulants at all SRTs were proteins and SMPs contributing to dominant cake layer formation. This study revealed a more stable reactor performance with a stable microbial community at the SRT of 25 days. However, the membrane flux permeability has declined at 25 days SRT. The findings of this study are useful for future AnCMBR design and process optimization for energy-efficient domestic wastewater treatment and reuse.

## Figures and Tables

**Table 1:** The operational conditions of an anaerobic yttria-based ceramic membrane bioreactor (AnCMBR) at different solid retention times (SRTs). HRT: hydraulic retention time, MLSS: mixed liquor suspended solids, MLVSS: mixed liquor volatile suspend solids, OLR: organic loading rate, TMP: transmembrane pressure.

Parameters	Stage 1	Stage 2	Stage 3
Time (days)	1–50	51–75	76–150
Effective volume (L)	15	15	15
HRT (h)	48	48	48
OLR (kg COD m^−3^ d^−1^)	0.23 ± 0.09	0.21 ± 0.05	0.29 ± 0.10
Cross flow rate (m/s)	2.48 ± 0.04	2.45 ± 0.04	2.53 ± 0.08
Backwashing (s d^−1^)	60	60	60
Temperature (°C)	31.6 ± 2.46	32.01 ± 1.53	30.5 ± 3.14
SRT (days)	100	50	25
MLSS (g/L)	9.08 ± 4.18	2.70 ± 0.50	1.45 ± 0.36
MLVSS (g/L)	4.37 ± 2.24	1.21 ± 0.31	0.84 ± 0.27
MLVSS/MLSS	0.46 ± 0.04	0.43 ± 0.04	0.58 ± 0.12
TMP (kPa)	73.33 ± 2.01	75.22 ± 1.18	76.54 ± 4.94
Flux (Lm^−2^ h^−1^)	50.82 ± 4.39	49.68 ± 2.38	34.40 ± 13.46
Permeability (Lm^−2^ h^−1^/kPa)	0.69 ± 0.06	0.66 ± 0.03	0.44 ± 0.15

**Table 2:** The composition of synthetic domestic wastewater.

Chemical	Concentration (g/L)
D (+) – Glucose monohydrate	200
Triptone	15
Calcium chloride dehydrate (CaCl.2H_2_O)	25
Potassium dihydrogen phosphate (KH_2_PO_4_)	17.5
Sodium hydrogen carbonate (NaHCO_3_)	137.5
Ammonium chloride (NH_4_Cl)	95.5
Sodium acetate (C_2_H_3_NaO_2)_	100
Magnesium sulfate (MgSO_4_.7H_2_O)	50

## Supplementary Materials

The following are available online at https://www.mdpi.com/2077-0375/10/9/196/s1, Figure S1: Automatic control strategy, Figure S2. The variation of operational temperature, pH, and ORP, Figure S3. The variation of (a) volatile fatty acids, (b) VFA/ALK ratio during the reactor operation, Figure S4. Transmembrane pressure, flux, permeability and CFV evolution at different SRTs, Figure S5. 3D-EEM spectra of influent, effluent, and extracted EPS and SMP at different SRTs, Figure S6 the principal components’ (PCs) loading plots at three SRT stages, Figure S7. Heat map at genus level for bacterial community responses at different SRTs, Figure S8. Environment factor correlation analysis by RDA for bacteria (a) and archaeal (b) community shifts, Figure S9. Genus-level heat map for archaeal community variation at different SRTs, Table S1. Fluorescence Spectral parameters of samples of influent, effluent, and extracted Anaerobic Sludge, Table S2. The dominant microorganisms and their functions during the study, Table S3. Bacteria Alpha Diversity indices, Table S4. Archaea Alpha Diversity indices.
