# Supplementary material for: Effects of Solids Retention Time on the Anaerobic Membrane Bioreactor with Yttria-Based Ceramic Membrane Treating Domestic Wastewater at Ambient Temperature
Source: Membranes (Basel). 2020 Aug 21;10(9):196. doi: 10.3390/membranes10090196 (PMC7559899; doi:10.3390/membranes10090196)
Supplement: Supplementary file 1 [file membranes-10-00196-s001.pdf]

Supplementary material

Effects of solids retention time on the Anaerobic Membrane Bioreactor with yttria-based ceramic tubular membrane treating domestic wastewater at ambient temperature

Rathmalgodage Thejani Nilusha[1, 2, 3,4,] Dawei Yu[1,3 ,4,]Junya Zhang[1,3,4,] and Yuansong Wei[1,3 ,4,5, *]

1. State Key Joint Laboratory of Environmental Simulation and Pollution Control, Research Center for Eco-Environmental Sciences, Chinese Academy of Sciences, Beijing, 100085, China; nthejani@yahoo.com (R.T); dwyu@rcees.ac.cn (D.W), jyzhang@rcees.ac.cn (J.Z)
2. Environment Technology Section, Industrial Technology Institute, 363, Bauddhaloka Mawatha, Colombo 07, 00700, Sri Lanka
3. Department of Water Pollution Control Technology, Research Center for Eco-Environmental Sciences, Chinese Academy of Sciences, Beijing 100085, China
4 .University of Chinese Academy of Sciences, Beijing 100049, China
5 .Institute of Energy, Jiangxi Academy of Sciences, Nanchang 330029, China

*Correspondence: yswei@rcees.ac.cn; Tel.: +86-10-6284-9690 (Y.W)

Number of Pages 12

Number of Figures: 9

Number of Tables: 4

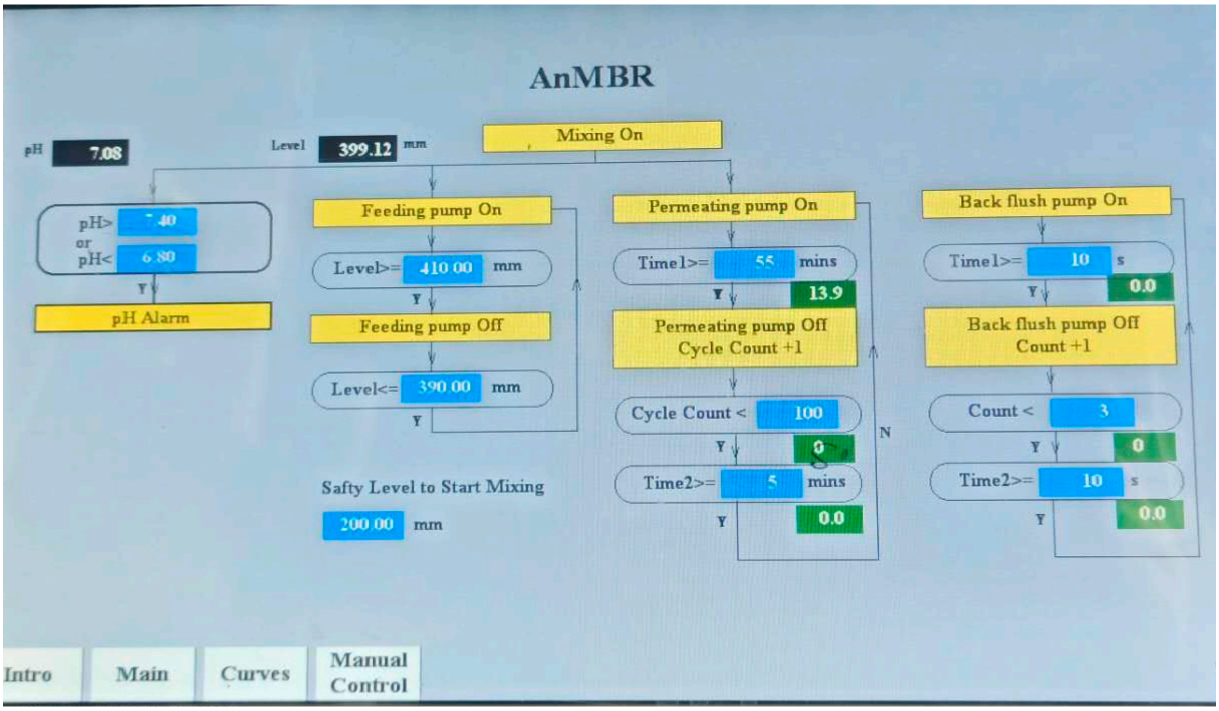

Figure. S1. Automatic control strategy

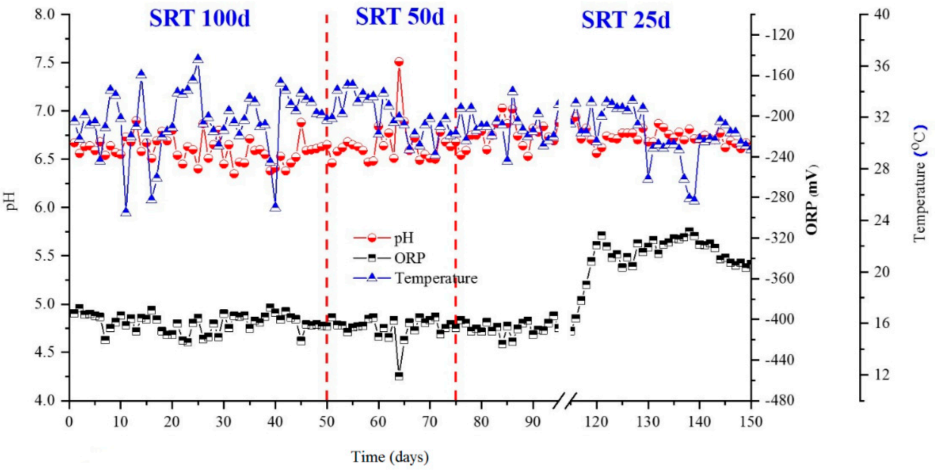

Figure. S2. The variation of operational temperature, pH and ORP

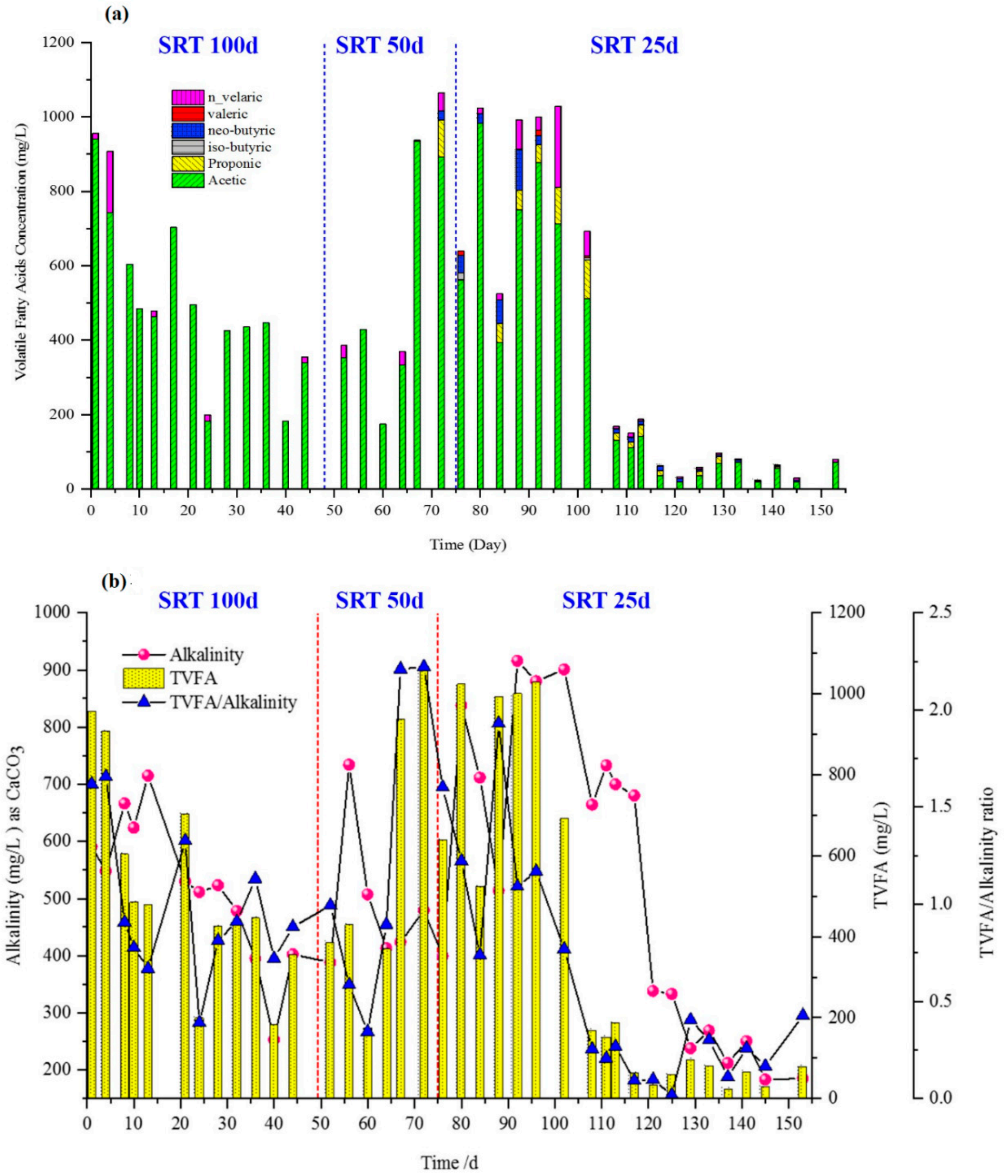

Figure. S3. The variation of (a) volatile fatty acids, (b) VFAs/ALK ratio during the reactor operation

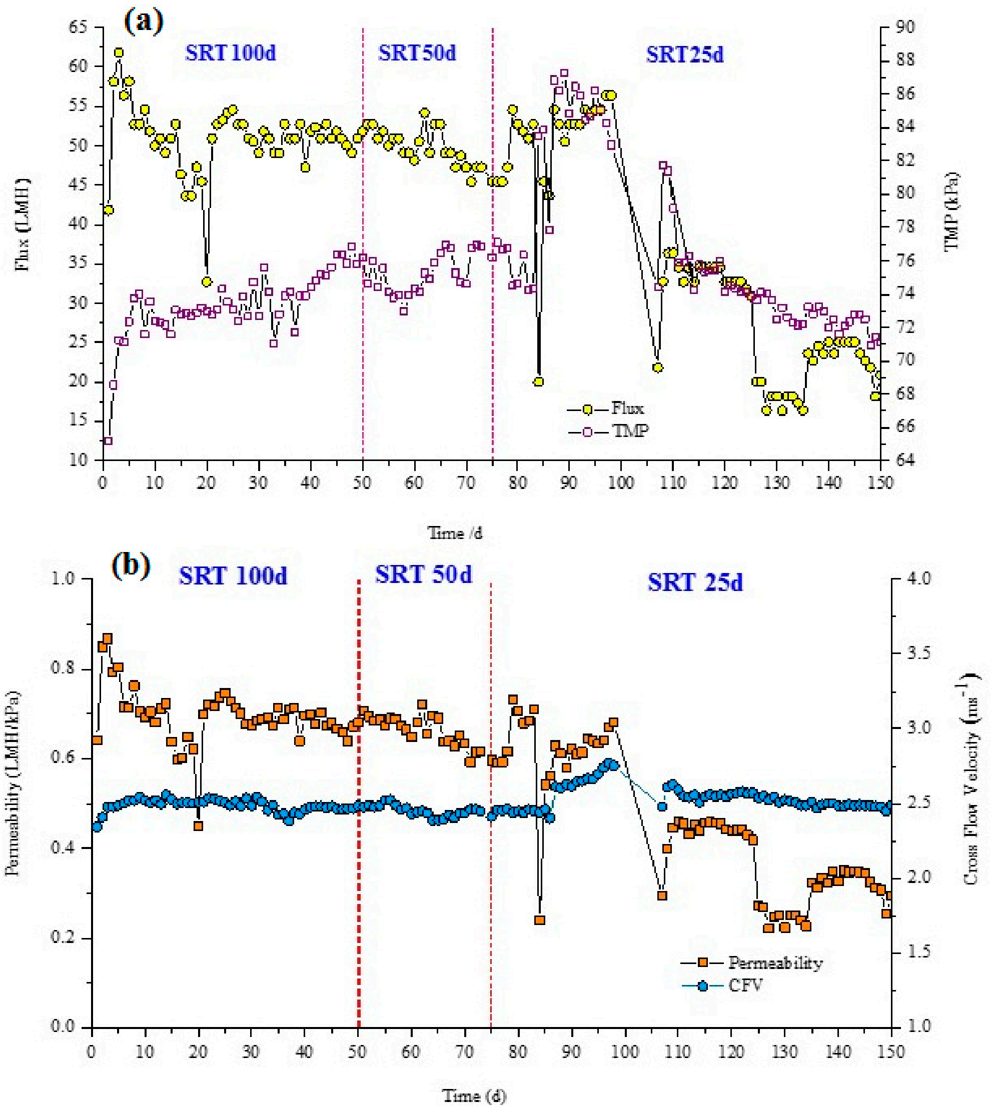

Figure. S4. Transmembrane Pressure, Flux, Permeability and CFV evolution at different SRTs

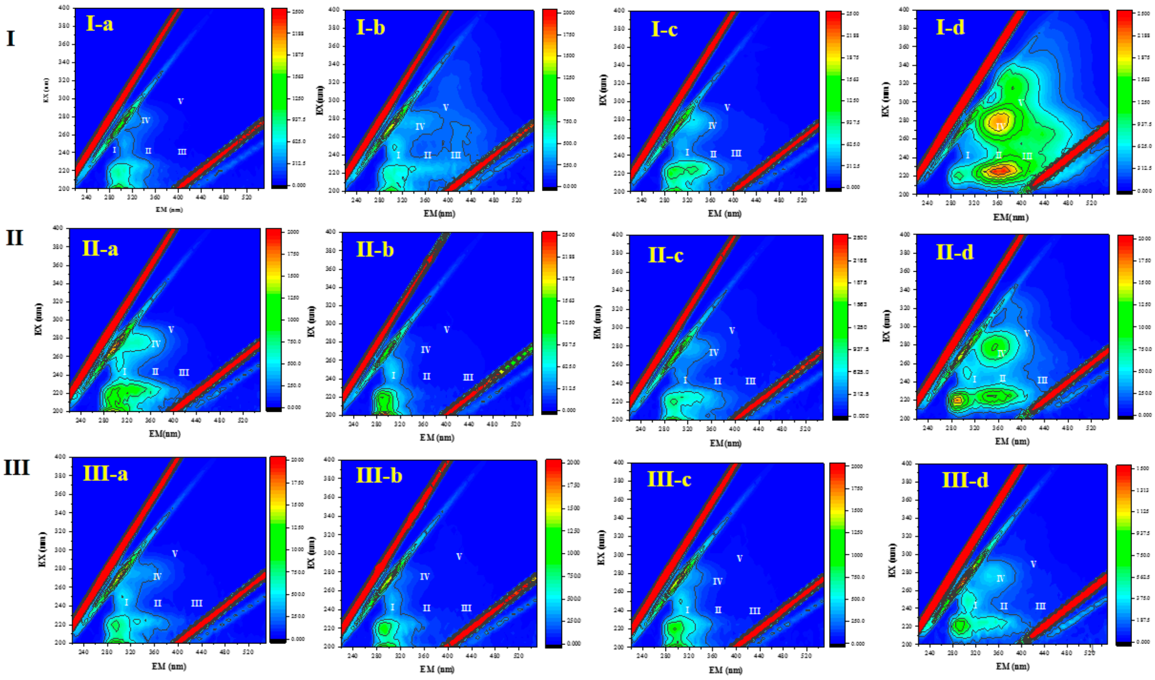

I-SRT 100days (I-a) Influent (I-b) permeate (I-c) AnMBR soluble fraction (I-d) AnMBR bound fraction
I- SRT 50days (II-a) Influent (II-b) permeate (II-c) AnMBR soluble fraction (II-d) AnMBR bound fraction
II- SRT 25days (III-a) Influent (III-b) permeate (III-c) AnMBR soluble fraction (III-d) AnMBR bound fraction
Region I: Tyrosine like proteins I; Region II: Tryptophan like protein; Region III: Fulvic acid-like (FA) substances; Region IV: Soluble microbial by-product-like substances; Region V: Humic acid-like (HA) substances

Figure .S5. 3D-EEM spectra of influent, effluent and extracted EPS and SMP at different SRTs

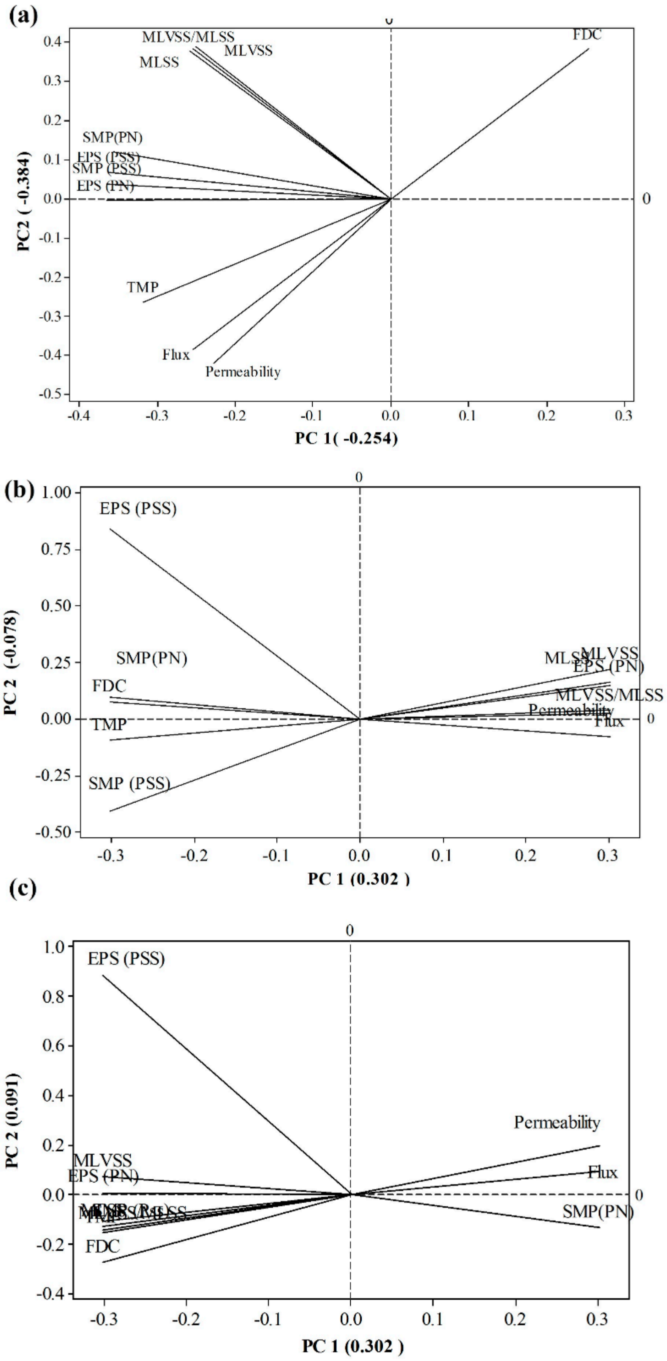

Figure .S6. The principal components' (PCs) loading plots at three SRT stages (a) SRT 100days (b) SRT 50days (c) SRT 25 days

	Seed 1	Seed 2	D01	D15	D30	D45	D60	D75	D90	D110	D125	D150
g__norank_f__Rikenellaceae	0	71	99	14595	26124	22575	13399	13092	9325	1899	1485	2337
g__Trichococcus	0	6	15304	3613	2617	3751	8680	10287	6566	7864	2725	7402
g__Raoultella	3	11	6679	10513	5548	3448	7904	3599	3000	759	520	1021
g__DMER64	0	4835	2	1	0	1	2262	4547	7181	5338	1845	4139
g__Chlorobium	0	0	0	0	0	0	1251	8977	5253	5066	662	4820
g__norank_f__Lentimicrobiaceae	3138	124	614	29	28	43	170	2268	1788	1082	5535	5749
g__Proteiniphilum	9512	573	4132	3512	899	171	146	43	28	7	1	16
g__norank_f__norank_o__D8A-2	9560	397	5356	507	83	95	197	158	1399	459	350	333
g__norank_f__Synergistaceae	0	8575	30	71	112	402	1209	373	1176	522	553	814
g__norank_f__Anaerolineaceae	866	3504	97	6	42	131	2842	352	1115	957	1766	1361
g__norank_f__norank_o__DTU014	6279	187	1375	33	148	1717	446	170	832	561	375	494
g__norank_f__M2PB4-65_termite_g	0	6140	2	181	61	114	3185	467	917	201	7	46
g__unclassified_f__Cloacimonadace	0	381	1	0	672	7277	1116	370	580	152	38	24
g__norank_f__Syntrophomonadacea	7978	14	2190	42	20	4	6	3	2	1	2	5
g__Aquaspirillum	0	1	1	0	0	0	43	56	5023	1394	1535	1713
g__Christensenellaceae_R-7_group	0	483	1094	1484	966	1056	1582	425	768	425	397	676
g__Defluviitoga	4816	5	2689	665	137	64	2	0	0	0	1	5
g__Longilinea	0	1685	45	86	528	322	1446	58	258	286	2318	1350
g__norank_f__Halothiobacillaceae	0	2	84	921	401	150	794	2359	1352	270	750	758
g__Enterococcus	2	3	6843	179	7	1	7	4	339	87	36	37
g__unclassified_p__Patescibacteria	0	0	0	0	0	0	0	0	769	863	1551	4335
g__norank_f__Prolixibacteraceae	0	236	10	438	876	1515	2198	606	661	329	207	381
g__norank_f__Spirochaetaceae	0	99	6	122	50	240	235	109	495	611	2809	2430
g__Pseudomonas	0	24	418	1843	58	263	67	49	294	1099	1253	1698
g__Desulfovibrio	1	113	1865	962	142	399	1081	394	972	330	290	420
g__Petrimonas	6	1303	379	747	1174	864	506	344	435	331	49	234
g__Candidatus_Caldatribacterium	1661	676	2991	427	133	52	138	6	59	22	75	61
g__Aeromonas	0	0	43	494	22	3	19	25	115	3004	76	2456
g__Acetomicrobium	3304	173	2146	241	107	17	11	0	1	0	5	5
g__Methanobacterium	0	4023	0	0	0	1	732	47	276	110	312	137
g__Arcobacter	0	611	1187	3020	27	6	107	9	12	77	186	313
g__norank_f__norank_o__LD1-PB3	3	839	2	0	0	1	837	205	1433	760	622	624
g__norank_f__norank_o__norank_c	1	12	237	968	745	1522	61	13	862	227	119	140
g__Keratinibaculum	4216	0	463	15	0	0	2	0	0	0	2	0
g__Geobacter	0	72	55	350	28	23	796	42	1057	735	628	827
g__unclassified_k__norank_d__Bac	167	57	46	16	33	76	259	30	87	61	2530	1243
g__Exiguobacterium	0	0	764	601	267	163	960	1012	520	65	2	45
g__unclassified_f__Anaerolineaceae	13	71	9	45	213	742	1367	89	227	150	889	492
g__Aminobacterium	0	3891	0	0	0	0	153	2	43	16	65	13
g__Methanosaeta	0	138	0	0	0	0	44	12	375	476	1815	1216
g__Acinetobacter	2	36	1065	2384	82	158	42	42	63	77	10	52
g__Caldicoprobacter	3187	8	729	60	13	0	3	0	0	0	2	0
g__Bacteroides	0	4	3	24	83	426	89	185	1857	475	319	392
g__unclassified_f__Burkholderiaceae	0	282	971	482	136	65	60	14	128	601	148	715
g__Flexilinea	0	1793	7	20	97	466	652	92	128	51	154	70
g__norank_f__Pedosphaeraceae	0	2852	3	0	2	115	322	1	145	24	22	9
g__Erysipelotrichaceae_UCG-004	0	11	1	17	157	385	380	226	1009	483	65	562
g__LNR_A2-18	0	2293	0	0	4	13	314	171	220	57	2	1
g__Sulfuricurvum	0	1	85	1314	3	55	64	8	12	11	732	577
g__Thiovirga	0	5	240	55	4	320	101	64	91	22	1239	682

Figure S7. Heat map at Genus Level for bacterial community responses at different SRTs

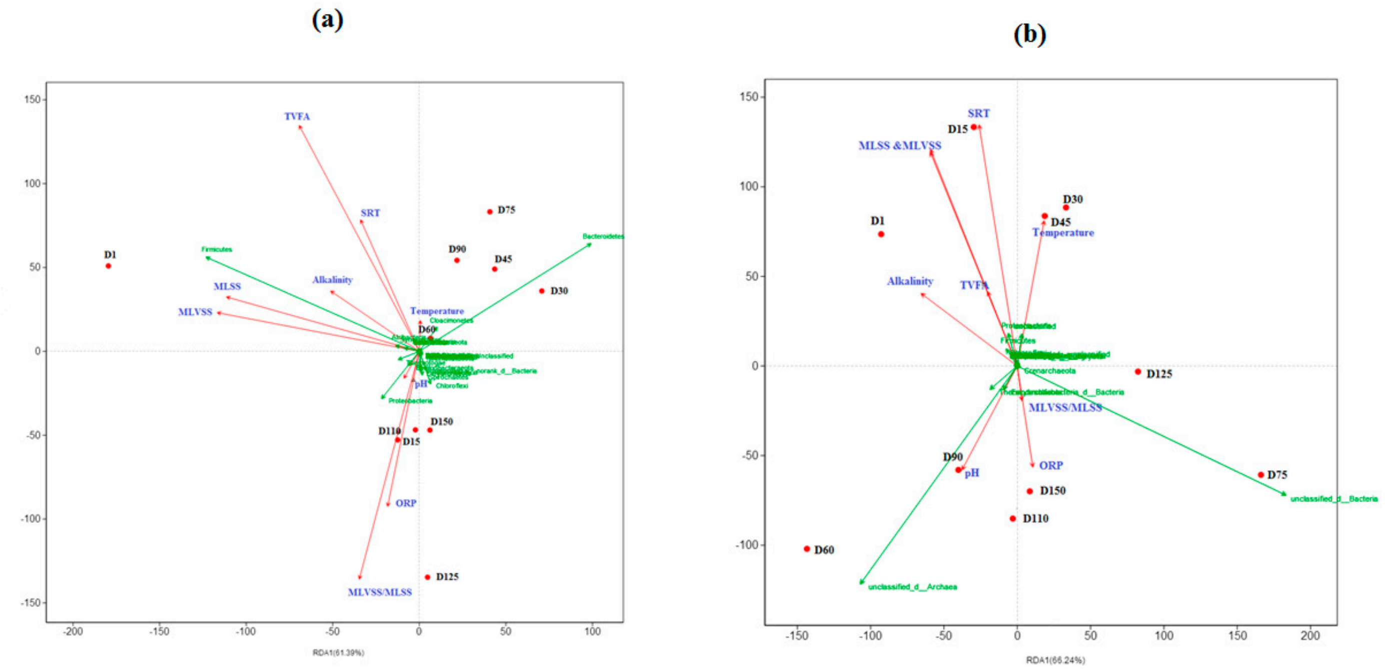

Figure .S8. Environment factor correlation analysis by RDA for (a) bacteria and (b)archaeal community shifts

	Seed 1	Seed 2	D01	D15	D30	D45	D60	D75	D90	D110	D125	D150
g__unclassified_d__Bacteria	21256	34326	12295	19643	34318	31543	15734	69142	31186	39972	49510	40942
g__unclassified_d__Archaea	26739	12849	14936	626	75	1122	40851	3373	25179	24666	4957	21296
g__Methanobacterium	0	7649	0	3	1	0	2803	97	3384	1030	236	621
g__unclassified	690	278	1027	926	10657	492	128	35	699	50	20	7
g__Thermodesulfobacterium	0	1	1	5	1	308	8280	15	13	25	104	40
g__unclassified_o__Methanosarcinales	0	117	3	730	288	44	1554	72	101	746	584	1455
g__unclassified_d__unclassified	1	3091	80	130	866	77	290	96	30	65	8	23
g__unclassified_c__Thermoplasmata	0	845	0	0	4	28	31	3	2	43	1281	1539
g__Klebsiella	0	3	797	2127	277	336	1	15	12	5	0	0
g__Trichococcus	0	0	1949	1092	40	74	0	23	0	17	0	2
g__unclassified_p__Verrucomicrobia	3	2741	9	88	51	0	8	0	9	9	1	1
g__Pseudomonas	36	59	342	2080	2	5	0	0	3	7	0	0
g__Enterococcus	0	0	1899	52	0	0	0	0	0	0	0	0
g__Arcobacter	0	82	124	853	1	0	0	0	0	0	0	0
g__Longilinea	0	0	13	727	147	40	1	98	0	1	0	0
g__unclassified_f__Planctomycetaceae	0	55	9	510	20	1	1	0	422	2	0	1
g__Desulfovibrio	0	27	179	625	27	96	1	0	6	4	0	0
g__Thermogymnomonas	0	55	0	812	13	0	4	1	1	0	0	2
g__unclassified_o__Clostridiales	341	60	280	108	14	0	0	7	1	0	0	0
g__Methanoculleus	308	16	57	310	55	4	15	2	1	12	5	10
g__Paracoccus_f__Rhodobacteraceae	0	0	424	152	0	0	0	0	0	0	0	0
g__Acetomicrobium	107	9	318	103	2	2	0	0	0	0	0	0
g__unclassified_f__Anaerolineaceae	0	276	0	53	90	9	6	65	9	2	0	0
g__Acinetobacter	17	44	75	318	4	10	0	0	0	0	0	0
g__Methanospirillum	0	1	0	0	0	0	60	1	4	128	66	153
g__Exiguobacterium	0	0	95	215	1	1	0	7	0	0	0	0
g__Victivallis	0	7	0	223	35	4	0	0	0	0	0	0
g__Tepidimicrobium	109	0	111	31	0	0	0	0	0	0	0	0
g__unclassified_p__Bacteroidetes	0	10	5	213	8	9	0	0	0	0	0	0
g__Enterobacter	0	7	104	99	5	0	0	0	0	0	0	0
g__Opitutus	0	0	0	0	0	0	3	48	158	0	0	0
g__Aeromonas	0	0	7	148	1	0	0	0	2	24	0	3
g__Syntrophomonas	19	60	28	47	0	0	0	0	0	0	0	0
g__unclassified_c__Thermoprotei	0	90	0	0	0	0	8	5	4	10	14	21
g__Acidovorax	0	0	0	147	1	0	0	0	0	0	0	0
g__Ensifer	0	0	113	23	0	0	0	0	0	0	0	0
g__Macellibacteroides	0	4	0	109	3	0	0	0	0	0	0	0
g__Bacillus_f__Bacillaceae	0	107	2	4	0	1	0	0	0	0	0	0
g__Vitis	17	64	9	7	3	13	0	0	0	0	0	0
g__unclassified_f__Ruminococcaceae	0	68	0	31	4	1	0	4	0	0	0	0
g__Propionicimonas	0	0	23	70	6	0	0	0	0	0	0	0
g__Desulfobulbus	27	0	70	2	0	0	0	0	0	0	0	0
g__Citrobacter	0	0	3	1	92	1	0	0	0	0	0	0
g__Christensenella	0	1	26	49	2	11	0	0	0	0	0	0
g__unclassified_p__Euryarchaeota	0	0	0	0	0	0	0	0	0	0	40	28
g__unclassified_p__Firmicutes	1	0	1	49	6	6	0	0	0	0	0	0
g__Methanothermobacter	3	0	3	49	0	0	0	0	0	0	0	0
g__unclassified_p__Candidatus_Latescibact	0	0	0	49	3	1	0	0	0	0	0	0
g__Methanobrevibacter	0	10	0	1	0	0	2	8	5	19	0	3
g__Geobacter	0	7	5	26	7	0	0	0	0	0	0	0

Figure S9 Genus level heat map for archaeal community variation at different SRTs

Table S1 Fluorescence Spectral parameters of samples of influent, effluent and extracted anaerobic sludge

Stage	Sample	Region 1 EX: 200-250 EM230-330	Region 11 EX: 200-250 EM330-380	Region 111 EX: 200-250 EM380-500	Region IV EX: 250-280 EM200-380	Region V EX: 280-500 EM380-500	FI index	BIX	HIX
SRT100	Influent (I-a)	EM295 EX200 Z1361	ND	ND	EM310 EX280 Z1666	ND	1.71	0.89	0.202
	Effluent (I-b)	EM300 EX200 Z 948.9	ND	ND	EM305 EX275 Z1683	ND	2.15	1.00	0.24
	AnMBR soluble (I-c)	EX220 EM295 Z1006<br>EX225 EM325 Z1017	ND	ND	EX275 EM310 Z873.8	ND	2.01	1.08	0.20
	AnMBR bound (I-d)	Ex220 EM290 Z1017	EX225 EM365 Z2342	ND	EX280 EM360 Z2113	ND	1.64	1.64	0.57
SRT50	Influent (II-a)	EX200 EM295 Z1687	ND	ND	EX275 EM305 Z1977	ND	1.59	1.19	0.10
	Effluent (II-b)	EX200 EM290 Z2404	ND	ND	EX275 EM305 Z1652	ND	1.73	0.97	0.139
	AnMBR soluble (II-c)	EM290 EX295 Z1027	ND	ND	EX280 EM310 Z1700	ND	2.15	1.73	0.24
	AnMBR bound (II-d)	EX220 EM290 Z1767	EX225 EM355 Z1302	ND	EX275 EM305 Z1644<br>EX355 EM280 Z1075	ND	1.59	1.75	0.26
SRT25	Influent	EX200 EM295 Z1145<br>Ex220 EM295 Z 1145	ND	ND	EX275 EM305 Z1690	ND	2.1	1.07	0.13
	Effluent	EX220 EM290 Z990.9	ND	ND	EX275 EM305 Z1572	ND	1.68	0.94	0.17
	AnMBR soluble	EX220 EM295 Z1050	ND	ND	EX 275 EM305 Z1673	ND	1.84	1.01	0.13

AnMBR bound	EX220 EM290 Z800.3	ND	ND	EX275 EM300 Z1112	ND	1.79	1.30	0.17

*ND :Not Detected

Table S2 The dominant microorganisms and their functions during the study

Bacteria (Phylum level)				
Phylum	***Function***	***Substrates***	***Product***	***Reference***
Firmicutes	Hydrolysis and hydrogenogenic acidogenesis	Propionate Acetate butyrate	Volatile fatty acids	[69]
Proteobacteria	hydrolysis and hydrogenogenic acidogenesis	cellulose, proteins, pectin	Volatile fatty acids	[70]
Bacteriodetes	Fermentative bacteria	organic materials	organic acids, CO_2 and H_2	[66]
Cloacimonetes	Propionate degradation	Propionate	Acetate	[69]
Synergistes	glucose- and acetate-utilizing bacteria	Glucose Acetate	CO_2 and H_2	[64]
Actinobacteria	Biodegradation of aromatic hydrocarbons	Aromatic hydrocarbons	Volatile fatty acids	[69]
Thermogates	syntrophically oxidize acetate	Acetate	CO_2 and H_2	[65]
Choroflexi	H_2-oxidizing homo acetogenesis	halogenated organics	H_2	[61]
Euryarchaeota	Methane-Producing Archaea	$H_2 + CO_2$, CO, formate, C_1-methylated compounds Acetate	CH_4	[71]
Spirochaetes	syntrophic acetate-oxidizing	Acetate	CO_2 and H_2	[72]
		**Archaea (Family Level)**		
Family	***Function***	***Substrates***	***Product***	***Reference***
Methanosarcinaceae	Methane production	carbon monoxide, carbon dioxide, and methylated C1 compounds) in addition to acetate	Methane	[73]
Methanobacteriaceae	Methane production	$H_2 + CO_2$, CO, formate, C_1-methylated compounds	CH_4	[73]
Methanosaetacea	Methane production	Acetate	CH_4	[73]
Methanomicrobiales	Methane production	$H_2 + CO_2$, formate, ethanol, 2-propanol, 2-butanol, cyclopentanol	CH_4	[73]
Methanosacinaceae	Methane production	Hydrogen	CH_4	[73]

acetate

Table S3 Bacteria Alpha Diversity indices

Sample\Estimators	sobs	shannon	simpson	ace	chao	coverage
Seed 1	30	0.877534	0.472152	38.169998	39.333333	0.999839
Day 1	89	1.664905	0.302695	95.023963	93.230769	0.999691
Day 15	107	2.044208	0.345301	108.942489	107.666667	0.999853
Day 30	67	0.851994	0.574718	75.631639	76.75	0.999725
Day 45	50	0.459317	0.841135	103.00987	113.333333	0.999416
Seed 2	90	1.467049	0.352368	95.41938	93.75	0.999842
Day 60	27	1.169061	0.409589	39.3039	32.25	0.9999
Day 75	31	0.258858	0.895649	31.871671	31.166667	0.999973
Day 90	29	0.996875	0.431559	34.297185	32	0.999902
Day 110	35	0.839767	0.493943	52.491587	41	0.999865
Day 125	17	0.526847	0.766169	17.679111	17	0.999982
Day 150	24	0.911396	0.487661	26.64125	24.5	0.999955

Table S4: Archaea Alpha Diversity indices

Sample\Estimators	sobs	shannon	simpson	ace	chao	coverage
Seed 1	291	3.462506	0.05852	303.966787	304	0.999618
Day 1	456	3.34318	0.086142	561.430706	588.717391	0.998357
Day 15	356	3.064635	0.113978	461.963293	476.731707	0.998216
Day 30	323	2.106304	0.345905	429.748134	465.676471	0.997814
Day 45	392	2.778037	0.182463	477.977872	502.534884	0.998259
Seed 2	664	4.245772	0.029664	779.766666	786.766234	0.997938
Day 60	692	3.824884	0.068945	849.222529	829.53125	0.997698
Day 75	685	3.876093	0.051158	880.534119	895.45	0.997332
Day 90	582	3.704362	0.067103	733.238556	733.075949	0.996483
Day 110	746	4.660758	0.021478	919.968502	963.2	0.996608
Day 125	768	4.338893	0.034709	959.760012	997.2	0.997129
Day 150	291	3.462506	0.05852	303.966787	304	0.999618

References

61.Riviere, D.; Desvignes, V.; Pelletier, E.; Chaussonnerie, S.; Guermazi, S.; Weissenbach, J.; Li, T.; Camacho, P.; Sghir, A. Towards the definition of a core of microorganisms involved in anaerobic digestion of sludge. ISME J. 2009, 3, 700–714, doi:10.1038/ismej.2009.2.

64.Bialek, K.; Kumar, A.; Mahony, T.; Lens, P.N.; O'Flaherty, V. Microbial community structure and dynamics in anaerobic fluidized-bed and granular sludge-bed reactors: Influence of operational temperature and reactor configuration. Microb. Biotechnol. 2012, 5, 738–752, doi:10.1111/j.1751-7915.2012.00364.x.
65.Greses, S.; Gaby, J.C.; Aguado, D.; Ferrer, J.; Seco, A.; Horn, S.J. Microbial community characterization during anaerobic digestion of Scenedesmus spp. under mesophilic and thermophilic conditions. Algal Res. 2017, 27, 121–130, doi:10.1016/j.algal.2017.09.002.
66.Ariesyady, H.D.; Ito, T.; Okabe, S. Functional bacterial and archaeal community structures of major trophic groups in a full-scale anaerobic sludge digester. Water Res. 2007, 41, 1554–1568, doi:10.1016/j.watres.2006.12.036.
69.Lin, Q.; He, G.; Rui, J.; Fang, X.; Tao, Y.; Li, J.; Li, X. Microorganism-regulated mechanisms of temperature effects on the performance of anaerobic digestion. Microb. Cell Fact. 2016, 15, 96, doi:10.1186/s12934-016-0491-x.
70.Ali, N.; Gong, H.; Liu, X.; Giwa, A.S.; Wang, K. Evaluation of bacterial association in methane generation pathways of an anaerobic digesting sludge via metagenomic sequencing. Arch. Microbiol. 2020, 202, 31–41, doi:10.1007/s00203-019-01716-x.
71Nordgard, A.S.R.; Bergland, W.H.; Vadstein, O.; Mironov, V.; Bakke, R.; Ostgaard, K.; Bakke, I. Anaerobic digestion of pig manure supernatant at high ammonia concentrations characterized by high abundances of Methanosaeta and non-euryarchaeotal archaea. Sci. Rep. 2017, 7, 15077, doi:10.1038/s41598-017-14527-1.
72.Zhou, H.; Brown, R.C.; Wen, Z. Anaerobic digestion of aqueous phase from pyrolysis of biomass: Reducing toxicity and improving microbial tolerance. Bioresour. Technol. 2019, 292, 121976, doi:10.1016/j.biortech.2019.121976.
73.Guo, J.; Peng, Y.; Ni, B.J.; Han, X.; Fan, L.; Yuan, Z. Dissecting microbial community structure and methane-producing pathways of a full-scale anaerobic reactor digesting activated sludge from wastewater treatment by metagenomic sequencing. Microb. Cell Fact. 2015, 14, 33, doi:10.1186/s12934-015-0218-4.
